# Negotiating technology-mediated interaction in health care

**DOI:** 10.1057/sth.2014.18

**Published:** 2015-01-28

**Authors:** Erna Håland, Line Melby

**Affiliations:** a Department of Adult Learning and Counselling, Norwegian University of Science & Technology (NTNU), Trondheim, Dragvoll, 7491 Norway; b SINTEF Technology & Society, P.O. Box 4760 Sluppen, Trondheim, 7465 Norway. E-mail: Line.Melby@sintef.no; c Institute of Health and Society, University of Oslo, P.O. Box 1130 Blindern, 0318 Oslo, Norway

**Keywords:** home care services, negotiations, technology-mediated interaction, work

## Abstract

The health-care sector is increasingly faced with different forms of technology that are introduced to mediate interaction, thus fully or partially replacing face-to-face meetings. In this article we address health personnel's experiences with three such technologies, namely: electronic messages, video conferences and net-based discussion forums. Drawing on Goffman's perspectives on interaction and frame, we argue that when technologies are introduced to mediate interaction, new frames for understanding and making sense of situations are created. These new frames imply new ways of organising and making sense of experience, and require *work* by the participants in the interaction. In this article, based on interviews from two Norwegian research projects, we investigate *health personnel's work to make sense of technology-mediated interaction in health care.* We discuss this work represented in four categories: how to perform in a competent manner, how to negotiate immediacy, how to enable social cues and how to establish and maintain commitment. Concluding, we argue that the introduction of mediating technologies redefines what is considered up-to-date, ‘good' health-care work and challenges health personnel to change (some of) their work practices and moves, as a result, far beyond simple interventions aimed at making work more efficient.

## Introduction

Health information technologies, like electronic patient records, have for several years been a crucial part of clinical practice. Currently, we also witness to the introduction of other types of information and communication technologies (ICT), not specifically designed for health care. In this article we address health-care personnel's experiences with three such technologies in Norway, namely: electronic messages (e-messages), video conferences and net-based discussion forums. The technologies are not new in and of themselves, but they are new to the work practices to which they are introduced, and new to the different user groups. So, what is ‘new' is not the artefacts in and of themselves, but their arrangements with other objects and activities (Barry, 2001). Consequently, little is known regarding how these technologies are understood and adapted within the health-care setting and how they will influence work practice.

ICT in health care has received increased attention in Norway lately, and is in particular actualised with a recent health-care reform addressing coordination and collaboration across the sector. The reform states that electronic communication should be ‘the normal way' of communicating within health care (Norwegian Ministry of Health and Care Services, 2009). Further, in Norway and internationally, there is a growing interest in different forms of telemedicine applications (Oudshoorn, 2008). These technologies all imply that face-to-face interaction is partly or fully replaced by technology-mediated interaction. The strong pressure for increased communication through ICT in health care implies that we need to better understand how health personnel experience and use these technologies.

The three technologies in our study are all technologies that mediate interaction. Where health personnel previously would interact face-to-face, or use a well-established mediating technology, like the telephone, they are now also supposed to interact using e-messages, video conferences and net-based discussion forums. These and other types of mediating technologies are necessary to communicate across space and time, and pervade our lives (Whittaker, 2003). In the workplace and in leisure time a number of social encounters between people are mediated by technology. Whittaker (2003) argues that we need to investigate whether, how and why mediated communication differs from face-to-face communication. With respect to this, Walther *et al* (2005) identify two perspectives regarding whether communication is affected by the absence of non-verbal cues in mediated interaction. Within one perspective, it is argued that mediation implies less meaningful and/or effective communication, while within the other it is argued that people adapt to the medium and compensate for the absence of non-verbal cues.

The introduction of new ICTs interferes with existing communication and coordination structures (Hasvold and Scholl, 2011), and mediated communication is often seen as a poor transmitter of crucial information, like emotions (Mentis *et al*, 2013), but can also support new forms of, and more extensive, communication (Newman *et al*, 2009; Price *et al*, 2012; Furukawa and Driessnack, 2013). Studies show that health-care personnel, or health students, may benefit from using technologies (typically video conferences) in their professional development and learning. Technologies may be effective for learning purposes and represent a potential for developing new professional communities (for example, Newman *et al*, 2009). However, technologies can only be successful when they make sense within the existing social relations which they are to function. This means that translation or re-invention of technologies into everyday context of use is needed (Webster, 2002; Allen, 2012).

This study is situated within the research field concerning the introduction and use of ICT in health care and the aim is to contribute to an enhanced understanding of mediating technologies in health care. Several authors have addressed how the introduction of ICT in health care has a profound effect on the organisation of health care (Webster, 2002; Heath *et al*, 2003; Timmermans and Berg, 2003; Håland, 2012; Lyngstad *et al*, 2013; Melby and Hellesø, 2014), professional practice and patients' experience of illness (Oudshoorn, 2011), and how implementation processes are challenging because of the complexities of health care (Robert *et al*, 2009, 2010). Few studies that address introduction of ICT in health care have looked at e-messages, video conferences and net-based discussion forums. As far as we know, none of these have investigated in detail the work involved when users negotiate how to communicate and collaborate via these new technologies. In this article, we explore how people relate to and experience different forms of mediating technology within health care. When different types of technology are introduced in interaction situations – in addition to, or replacing, the physical face-to-face meeting – the technology both enables and impedes different forms of interaction. People interpret and negotiate the new possibilities and limitations offered by the technology and by this create new practices.

Drawing on Goffman's (1972, 1974, 2005) perspectives on social interaction, we argue that people work to maintain a more or less stable order for interaction in social situations. When technology is introduced to mediate interaction this creates a new *frame* (Goffman, 1974) for understanding and making sense of the specific situation. This new framework implies new ways of organising and making sense of experience, and requires *work* by the participants in the interaction. Health personnel negotiate frame – how to understand and participate in mediated interaction. In this article, we investigate *health personnel's work to make sense of technology-mediated interaction in health care.* We analyse the impact of these new frames on interactions and the efforts by participants to change and sustain them (Goffman, 1974). In order to understand technology as a mediator, we follow Wyatt *et al* (2008) who draw on Latour's (2005) distinction between an intermediary and a mediator. An intermediary is someone (something) who transports meaning or force without transformation. Mediators, on the other hand, transform, translate, distort and modify the meaning of the elements they are supposed to carry (Latour, 2005, p. 39). We acknowledge that the technologies in our study have the role of mediators; they have the potential to change the meaning of the information being transmitted, and alter the relationship between the communicating partners.

Our article builds on material collected in two different research projects in Norway. Both projects have addressed the introduction of ICT in municipal health care. Project A has studied public health workers' use of video conferencing and net-based discussion forums for competence development, while project B has looked at the introduction of e-messages in home care and at general practitioners (GPs) as a means of communication. Study designs, settings and methods are described in more detail in the Methods section. Even though the technologies are different, they have in common that they make users switch from face-to-face interactions, or telephone conversations, to different types of technology-mediated interactions. Hence, users encounter many of the same challenges and must learn to collaborate by the use of technology.

## The Interaction Order, Frame and Mediated Interaction

Goffman's concern was to study the elementary processes that are fundamental to all social life, and by this establish a new and specific field of study within sociology, namely the interaction order (Jacobsen and Kristiansen, 2004). He was interested in focused gatherings/encounters, and it is the myriad of these encounters, he claims, that constitutes the interaction order. Encounters are examples of what Goffman (1972) identifies as focused interaction, that is, what happens when people agree to maintain a single cognitive and visual focus within a limited time frame, for example, in a conversation.

The interaction order refers to the order and structure that are to be found in social situations where people meet face-to-face. In these meetings, an order that follows its own rules develops, as well as structures that ensure exchange and communication between people in these situations. Even if Goffman was mainly concerned with interaction that takes place face-to-face we will argue that his perspectives and concepts are relevant for understanding technology-mediated interaction. We follow Pinch (2010) in arguing that technologies are present in the interaction order and play a crucial part in how interaction is mediated, as the interaction order is ‘embedded within, mediated by, and staged by material circumstances and mundane technologies' (p. 419).

The self is, according to Goffman, first and foremost motivated by a desire to be well-regarded – and encounters offer possibilities for eliciting that regard (Best, 2005). Face-to-face interaction happens in and because of the presence of others, where visible signs of orientation and involvement in the situation are glances, gestures, positionings and verbal statements that people feed into the situation, either intended or unintended (Goffman, 2005). In *On face-work*, Goffman (2005) studies the face-work people engage in to maintain their own face and the face of others in the interaction. Face is understood as the positive and symbolic value a person attaches to himself and others attach to him. Further in this essay, Goffman underlines how important it is for a person to save one's own face and describes that the safest way of preventing threats to his face is to avoid contacts where it is likely that this could occur. Goffman is mostly concerned with immediate encounters, but also mentions mediated encounters (for example written expressions). However, he emphasises that it is through direct personal contact that the unique informational conditions prevail and where the importance of face is particularly visible. When the possibility of spoken interaction arises, a system of practices, conventions and rules come into play that guides and organises the exchange of messages in the situation. For example, when a person sends out a message, the other participants in the encounter are obliged to show that the message is received and that the content is either acceptable for all involved or that it can be contradicted in an acceptable manner. In *Alienation from interaction*, Goffman (2005) emphasises again the unique qualities of a conversation – a conversation creates a world and a reality for the participant where there are also other participants present. Further, he points to the importance of the many small signals in the interaction: ‘When individuals are in one another's immediate presence, a multitude of words, gestures, acts, and minor events become available, whether desired or not, through which one who is present can intentionally or unintentionally symbolize his character and his attitudes' (p. 114).

Goffman (1974) did not claim that interaction happens in a vacuum, in fact he devoted a 576 page essay to explore what surrounds an interaction – to *frame*. Frame refers to the relational dimensions of meaning, how participants in interactions organise experience and make sense of events. It guides the response to the question ‘what is it that's going on here'? Given the participants' understanding of what it is that is going on, they adjust their actions in relation to this understanding (Goffman, 1974). Goffman's concept is related to concepts like context, setting and background and concerns frameworks of interpretation, the frame participants interpret events within. Frame implies ‘guided doings' – participants are subjected to rules and expectations, to social appraisal of his or hers actions (Goffman, 1974, p. 22). Of particular interest to our study, is the importance Goffman makes of the *struggle* to make seemingly effortless actions work. He claims that competent performances are achieved through a process of negotiations ‘in a cold sweat' (Goffman, 1974, p. xii). This implies that participants have to *work* to make sense of a situation and to negotiate its meaning and its implications for action. When new technology is introduced in interaction situations, this implies new frames – new schema for the interpretation of what it is that is going on and new efforts by participants to make sense of the situation and how to act in an appropriate manner.

Several researchers have used the work of Goffman to investigate mediated communication, and in this article we will draw on a study of mobile telephone use (Rettie, 2009) in particular. One of the main arguments in Rettie's study is that we need to understand the difference between face-to-face interaction and mediated interaction, and to understand the difference between various *forms* of mediated interaction. There are differences between synchronous and asynchronous mediated communication; the study explores the boundaries of synchrony. Synchronous mediated communication can have characteristics that make it similar to a face-to-face encounter, as Goffman understands it, that is, an encounter where the participants cooperate to maintain focused interaction (Rettie, 2009). Even though the participants are not physically present in the same place in a telephone conversation or a video conference, they share the same time frame and a mediated co-presence – a mediated encounter (Rettie, 2009). In a video conference the participants can see, hear and talk in real-time, and this gives the participants the possibility to coordinate their actions and cooperate on the interaction. In asynchronous communication, like e-messages, on the other hand, this common, shared time frame does not exist, implying that it is more difficult for the participants to coordinate their actions (Rettie, 2009). A net-based discussion forum can have characteristics in line with what we find in synchronous mediated communication, but that requires that the participants agree to ‘meet' in the forum on specific points of time. Otherwise, the use of net-based discussion forums can be an example of asynchronous mediated communication, where the participants are not present in the same time frame.

Synchronous communication offers the participants a possibility to share an experience in real-time – ‘their shared practice of coordinated interaction creates the inter-subjective experience of a mediated encounter' (Rettie, 2009, p. 426). Synchronous media afford this possibility of interaction. Asynchronous media ‘does not afford continuous cooperative practice and the experience of a moment-by-moment intersubjectivity in a shared social reality' (Rettie, 2009, p. 126). The participants in this type of communication have a possibility to respond to each other's expressions, but there is no cooperation in real-time.

Rettie's (2009) study of mobile telephone use illustrates that the border between synchronous and asynchronous communication can be blurred and that it is not the technology itself that defines this border. SMS, e-mail and chat (for example via Facebook) all have the same transmission time, but expected time used for responses are very different and formed by normative expectations and user practices. E-mail was, in this study, perceived as rather asynchronous, while SMS was perceived as ‘near-synchronous.' It was acceptable to wait several days for a response to an e-mail, while a response to a SMS was expected quickly. A delayed answer to a SMS was perceived as sending a meaningful message, that is, an intended delay/no answer. The study shows that the experience of mediated interaction is ‘shaped both by the temporal characteristics of the medium and by normative framing expectations' (Rettie, 2009, p. 436). As Hutchby (2003) says: ‘there is a complex interplay between the normative structures of conversational interaction and the communicative affordances offered by different forms of technology'(p. 585).

In this article we use Goffman's (and Rettie's) concepts of interaction and frame to understand the work involved when health personnel experience and make sense of the various forms of mediating technologies (e-messaging, video conferences and a net-based discussion forum). We explore the negotiations taking place in their efforts to make sense of the new framework.

## Methods

This article draws on material collected in two different Norwegian research projects. Both projects used an explorative approach with open-ended interviews. This approach is well-suited for investigating the participant's own experiences of a phenomenon (Silverman, 2001). The Ombudsman for Privacy in Research (Norwegian Social Science Data Services) approved both studies.

In project A, we investigated the introduction of video conferences and a net-based discussion forum for competence development of health personnel in home care (nurses and assistants in home care and nursing homes) in a Norwegian region with large geographical distances. We conducted group interviews and individual interviews with 14 of the total 19 participants in the course ‘Ageing on the Internet' in 2011. The course aimed at strengthening the health-care workers' competence on elderly people and on how to use ICT for cooperation and learning at a distance. The interviews were taped and transcribed verbatim by the first author.

In project B, we investigated the introduction of e-messaging between home-care services and GPs. The e-message system was developed as a module that can be integrated with the different electronic patient record systems in use in Norway, and the aim was to better facilitate (electronic) communication between health-care workers across organisations. In 2008–2009, an e-messaging pilot was implemented in six municipalities, of which two were selected for this study. These were strategically chosen because the involved GPs and home-care nurses had the most experience with the use of e-messaging. We conducted interviews with 43 persons in total: 23 nurses, 11 GPs, 5 secretaries and 4 project managers. The interviews were a combination of individual interviews and group interviews and were conducted during 2011. The interviews were taped and transcribed verbatim by a research assistant. (Table 1)

### Analysis

Projects A and B were developed independent of each other, but were both connected to research activity at The Norwegian Centre for Electronic Patient Records. We discovered that even if the projects had different points of departure, they bore many similarities related to how health-care personnel work to make sense of technology-mediated interaction. Within both projects a rough thematic coding of the data had already been conducted before starting on the common analysis for this article. The current analysis has been conducted in close collaboration between First author and Second author. The data were analysed using an interpretative and eclectic approach, described by Kvale (2007) as ‘bricolage,' in which the aim was to generate meaning and see connections across the material. In an iterative process, we started out with discussing similarities and differences in how our interviewees had described their efforts to comprehend the technology as a tool for communication. Following the discussion, and introducing Goffman's perspectives to guide the analysis, we returned to our material to consider the suggested categories. This process was performed several times. We aimed at developing categories that were common for both projects and which, in our opinion, said something more general about technology-mediated interaction. Based on the interviewees' own expressions of their efforts to make sense of technology-mediated interaction, four categories which represented themes prevailing to a large extent in both projects, were developed. They are presented in the following section and exemplified with quotes from both projects.

## Making Sense of Technology-Mediated Interaction in Health care

When new technologies are introduced in interaction situations in health care, health personnel have to figure out how to relate to these technologies and how to make sense of them in their particular context. This involves continuous work and negotiations on a detailed level. In this article, we explore and discuss this work in relation to four categories that were particularly visible in our empirical material. We bring forward health personnel's own expressions of how they relate to new technologies in these situations. First, we investigate health personnel's efforts to make sense of how to perform in a competent and professional manner in technology-mediated interaction. Second, we explore how health personnel reflect upon how and when to communicate in these situations, how they negotiate immediacy. Third, we discuss how health personnel relate to a different and limited repertoire of social cues in technology-mediated interaction. Last, we look into what kind of work needs to be performed in order to maintain a mutual commitment between actors in technology-mediated interaction.

## Performing Competence

The introduction of technology-mediated interaction in addition to, or instead of, face-to-face interaction establishes new frames for addressing competence. To perform in a competent and professional manner then includes using new technology adequately. This represents work for all health personnel, and for some groups, it represents a major challenge. Some groups of health personnel in our study had never sent or received an e-mail, never opened an attachment, never used Power Point to present something and barely been on the Internet. Then being ‘forced' to use video conferences and to participate in a net-based discussion forum implied a great effort. These groups of health personnel can provide good care to patients and have a strong professional identity, but are now faced with a reality where they no longer can avoid using technology to perform in a competent manner. This might represent a threat to their ‘face,' as described by Goffman (2005), a threat to their positive and symbolic value. Goffman (2005) argues that the safest way to prevent threats to one's face is to avoid situations where that could happen, and some interviewees (in both projects) reacted by withdrawing from the situation. Others embraced the new situation and, through a lot of hard work, experienced to cope with the new demands in a larger extent:

[For us that] has not even turned on a computer before, it's fun. To participate in the discussion forum and send e-mails and send in assignments and download and cut and paste and everything. It really is. Because that we *have to* be able to do, that I've been told. (Project A, Participant 1, Health-care worker)

The health-care worker articulates how she has overcome a major barrier using technology and how this is experienced as something positive. She also acknowledges that using technology in these situations represents new demands that they, as health personnel, simply have to cope with. Using computers on a basic level is often regarded as something everybody copes with in our modern society, but our study shows that there are still groups where this represents a major challenge. Non-users of technology must still be reckoned with (Wyatt *et al*, 2002).

When the first barrier of simply learning the required technical skills is overcome, work still remains on a very detailed level, when health personnel try to make sense of the new technology. In a group interview, reflecting on a discussion from a physical seminar the day before, two interviewees explain:

Interviewee 1: It was a little interesting what was said yesterday about how to communicate online. That about smileys or no smileys, how it's perceived what you … Interviewee 2: … put out there … Interviewee 1: To have a section [in the course] about ethics, to have that beforehand, before people start to write … what can be commented on and what definitely not need to be posted in a net forum. Interviewee 2: It's so new, I guess, that they hadn't thought about that, but it's clear … one should think carefully. Interviewee 1: Before you post something, yes. Interviewee 2: Because there's no delete-button, that we wanted! [laughter] (Project A, Participants 1 and 2, Health-care workers)

The health-care workers articulate how they feel insecure expressing themselves in a net-based discussion forum and that they need training to do this, even down to the very detail of when to use smileys, and so on. A net-based discussion forum is often presented as an arena for informal, oral communication. However, since communication happens through writing and remains on the screen, it is difficult to withdraw unfortunate formulations, as one can do in oral communication. The health-care workers' wish for a ‘delete-button,' even if it is said jokingly, shows that they are insecure regarding what should or should not be written in this forum and how this arena for communication should be interpreted. Following Goffman's (1974) concept of frame, it is unclear within what frame the communication should be understood. Is it a situation with ‘oral' informal communication in a private setting, where one can ‘say' things that are not always completely thought through, or is it a more formal and public situation where one has to be precise in what is ‘said'? How do you perform in a competent manner in this setting? Some of the participants in the net-based discussion forum also used the text from the discussions, using cut and paste, as a point of departure for the written assignment they had to hand in, adding even more uncertainty regarding type of communication. Frame implies ‘guided doings,' meaning that participants in the interaction are subjected to rules and expectations (Goffman, 1974). In this setting, the rules and expectations are still unclear – the participants have to work to make sense of this situation and to negotiate its implications for action.

The introduction of new technology can also imply that particular parts of what health personnel have learnt in their formal training is no longer valid regarding how to perform in a competent and professional manner. Health personnel using e-messages experienced that what they had learnt in their formal training regarding how to relate to people did not always apply to these new situations where they were communicating via technology:

In training we learnt that we should be nice and write ‘with best regards' and our name below. But I can see that when I send a message, my name appears at the top [of the text] as sender. So after a while I thought it was so stupid to write ‘with best regards' when I saw my name anyway. So I stopped. (Project B, Participant 1, Home-care nurse)

The quote exemplifies how ‘traditional' ways of addressing people in formal communication is challenged with the introduction of e-messages. Moreover, here there is uncertainty regarding frame (Goffman, 1974). It is unclear if the e-messages should be understood as a ‘letter' between participants in a communication, then following expected rules regarding formal communication, or if they should be understood as pieces of information intended for the patient record. Shortly after the introduction, health personnel expressed that they also used to write, for example, ‘have a nice weekend' and add smileys to the messages, thus performing, as they would do in telephone conversations, before. However, after they had used e-messages for a longer period and after they had realised that the messages became part of the patients' record, they stopped adding this type of information. So, what we see is that the rules and expectations for how to perform in a competent manner in this setting are negotiated through a process that involves work on a very detailed level.

## Negotiating Immediacy

Health personnel experience that technology-mediated interaction differs from face-to-face and telephone interaction in several ways. A common theme, identified among the interviewees in both Projects A and B, was how health personnel struggle to define how to relate to time in technology-mediated interaction: How and what should be communicated at what time? How long is it acceptable to wait for an answer? Rettie (2009) shows that the borders between synchronous and asynchronous communication can be blurry in mediated interaction and that expected time used for response are influenced by normative expectations and user practices.

In project A, even though the net-based discussion forum affords ‘near-synchronous' communication (Rettie, 2009), it is not used in this way by the participants. They did not agree upon a time to meet and discuss in the forum, which means that the communication there is experienced as more asynchronous, less immediate and more split up:

In the discussion forum I thought … it wasn't that easy because … if you post something there, it could take days before you got any response, so then you didn't get any further … [contrary to] when you talk to someone, then you get feedback at once, so it's … maybe … if you had known them a little better, then you could have sent an SMS in addition – can you check, I have sent you something, so … (Project A, Participant 3, Health-care worker)

The health-care worker articulates how communicating in a net-based discussion forum is very different from face-to-face communication and that she does not receive the immediate response that you do face-to-face. Goffman (1972, 2005) highlights the unique qualities of a face-to-face encounter, where people agree to maintain a single cognitive and visual focus within a limited time frame. When various forms of mediating technology replace the physical encounter, the participants need to negotiate what is an appropriate time for response. The frame (Goffman, 1974) is changing, meaning that the participants have to negotiate if this is more or less synchronous communication, implying an immediate response, or if it is asynchronous communication, where it is acceptable to wait days for a response. The health-care worker in the quotation above reflects upon her own use of this particular technology, that they could have texted each other to get a more immediate response, thus signifying the need for negotiations to take place to make sense of and use this technology.

Health personnel using e-messages experienced that sometimes they had to wait for a long time for answers to the messages, even if the guidelines stated that replies should be given within three days:

I experience that it may take a long time before you get an answer. I just checked, and it was 14 days back [that we sent a request]. Some answers immediately, whilst with others you have to send a new request or call them on telephone and ask if they have seen the e-message. (Project B, Participant 2, Home-care nurse)

The nurse describes how she often has to wait for a long time for a response to the messages she sends and that the response time varies between the different persons she communicates with. Also, several nurses described that with persons who normally would respond quickly, they would expect a quick response. This shows how the expected response time is formed by normative expectations and user practices (Hutchby, 2003; Rettie, 2009), implying that there are negotiations on a very detailed level between the different groups and persons who are supposed to use the e-messages to collaborate. They have to negotiate when and how to use e-messages and what is an appropriate response time. In home care, because of the often experienced long response time from GPs, they would never use e-messages for urgent matters.

## Enabling Social Cues

A third category identified in our empirical material concerns health personnel's experience of reduced access to social cues in technology-mediated interaction, compared with face-to-face and telephone interaction, and how this challenges communication. Social cues refer to all the small signals people give – conscious or not – during interaction, signals that convey meaning and help people to interpret a conversation (Goffman, 2005). When introducing technology-mediated communication, many of the social cues implied in seeing and hearing each other are lost (cf. Walther *et al*, 2005; Mentis *et al*, 2013), or they have to be ‘reinvented,' given a new shape, adapted to the technology.

In both projects A and B our interviewees talk about difficulties in making communication and interaction ‘flow,' because of the lack of social cues. On a general level, for example, some health-care workers talked about how they experienced it to be much easier to talk than to write. They considered the net forum to be an obstacle rather than an enabler for smooth communication in their group work discussions. More specifically, interviewees expressed that technology-mediated interaction made it more difficult to detect the communication partner's mood and to make a joke, and it was easier to be misunderstood when you had to write instead of talking. In an interview with health-care workers in project A, this is exemplified in the following:

Health care worker: The disadvantages [in the net-based discussion forum] must be … maybe, that you don't see … the communication … you don't see if a person is mad or happy or … Interviewer: That you don't see the face? Health care worker: Yes. There can quickly be misunderstandings. Interviewer: Because it's written? Health care worker: Yes. You can't see … Interviewer: If it's a joke or … Health care worker: Yes. (Project A, Participant 4, Health-care worker)

The health-care worker describes how the fact that you cannot see each other creates possibilities for misunderstandings in the communication. The same concern is expressed by a GP in Project B, who had used e-messaging for a period of time:

It's like with e-mail; if you have an unfortunate formulation, it might be comprehended completely wrong by the recipient, and it might escalate … So, sometimes it's much simpler, just picking up the telephone. (Project B, Participant 3, GP)

The GP explains how e-mail and e-messages imply the possibility for misunderstandings, and that he sometimes actually prefers to use the telephone instead. The telephone is a well-established mediating technology where the fact that you can hear each other gives the communicating partners access to a richer repertoire of social cues.

The introduction of technology-mediated interaction means the introduction of new frames (Goffman, 1974) for health personnel. A reduced repertoire of social cues – or a need to reinvent relevant social cues – is part of the new frames. Health personnel negotiate how to adjust their communication and actions within the new frames. Some of the health personnel in Project A, using the net forum, even asked for more training in how to behave and express themselves in the forum to avoid misunderstandings – thus implying that the lack of social cues were considered challenging. However, we do not argue that the introduction of technologies that imply less access to social cues is one-sidedly negative for interaction. In Project B, some nurses explained that actually being able to sit down in peace and quiet instead of meeting GPs or having telephone conversations with them meant that they wrote, according to themselves, more well-considered and precise messages. This indicates that sometimes it is beneficial for communication to switch from an interaction form rich with social cues to one with less social cues. In particular, it can be efficient when exchanging information where precision and details are important and there exists a language for describing the matter of case (for example, reporting a patient's blood pressure values) and the sender's frame of understanding is less relevant.

What we see here, is that health personnel's efforts to make sense of social cues in technology-mediated interaction imply that they negotiate the frame of the interaction: does the interaction represent a formal exchange of information, meaning access to social cues might be less relevant? Or does it represent a conversation close to what is expected in informal face-to-face interaction, meaning a reduced access to social cues might be challenging? These negotiations take place on a daily basis and involve continuous work to establish a common frame of reference.

## Establishing and Maintaining Commitment

How to develop and maintain commitment to the technology and to your communication partners in technology-mediated interaction is the last category we will discuss in this article. In our empirical material we find examples of work that needs to be done and work that is done in order to establish and maintain commitment in technology-mediated interaction. The work necessary to facilitate participation and commitment also requires work by those other than the end-users, for example by management.

In Project A, interviewees had ideas about how to facilitate participation and commitment in a net-based discussion forum. When asked about possibilities for improvements, two interviewees suggested:

Interviewee 1: There should be some more information. Interviewee 2: Before the course, yes. Interviewee 1: I think maybe that the employer should take that part, then, because it was clear that there were some demands that people maybe didn't know about. So there should maybe be more information about how to work in groups. Interviewee 2: Online. Interviewer: How that works? Interviewee 1: Yes. Or, that you actually commit yourself even if you don't sit next to each other, that you have to commit, to go in[to the forum] and participate. (Project A, Participant 1 and 2, Health-care workers)

The two health-care workers point to the need for more information, better arrangements from the employer to facilitate participation, and a consciousness raising that participating in a net forum means committing to take part. In Project A, participants also raised the issue of ‘free riders' in the net forum. They argued that all participants should be active participants, and not only observe what the active actors did. This illustrates that the introduction of technology-mediated interaction creates a need for work to be done – to facilitate commitment – but that the participants have not yet established how this should be done or who is responsible for doing it. As Pinch (2010) points out, new technologies create new interactional problems that the participants in the interaction need to solve. From Goffman's (1974) perspective, we can say that a more coherent view of the technology and the interaction, a common frame, needs to be negotiated among the participants in order to establish how it is appropriate to commit to each other within this new frame.

The question of commitment was also an issue in Project B. Here we found examples of work when actors tried to make their communication partner commit to the interaction. In this project many nurses in home care were eager users of the e-messaging system, while some GPs used it infrequently. This imbalance caused problems in communication. For nurses the solutions could be to make a telephone call to the GP office, but also formal systems were developed to deal with discrepancies from the communication guidelines:

Some [doctors] are really quick, while others use a lot of time. And then you have to send a message, a so-called ‘exception message.' And if you don't receive a reply, you have to call the secretary, and you might be told that the doctor isn't present, or is somewhere else. And then you get your explanation on why you didn't receive a reply. (Project B, Participant 2, Home-care nurse)

Nurses in Project B would occasionally send discrepancy reports to the municipal administration. In this respect their work represents an educating effort *vis-à-vis* the less committed users, thus being part of the negotiations taking place in order to establish rules and expectations within a new frame (Goffman, 1974).

There is much work needed to make actors commit to participate in technology-mediated interaction, including promoting the use of technology in an appropriate and timely way. In this respect we see links back to the categories on performing competence and negotiating immediacy. Being committed to the interaction – and being a ‘good' communication partner – means first of all starting to use the technology, and thereafter negotiating what should encompass ‘correct' use.

## Conclusions

The health-care sector is increasingly faced with different forms of technology that are supposed to mediate interaction, thus fully or partially replacing face-to-face encounters (like for example many telemedicine initiatives). The rationale behind the introduction of these technologies is often to reduce costs and to enhance efficiency, but potential gains cannot be expected to be automatically realised. As we have shown in this article, there remains work to be carried out. Technologies that can be understood as mediators influence the meaning of the elements they are supposed to carry (Latour, 2005), implying that face-to-face interaction cannot simply be replaced by technology-mediated interaction without involving active sense-making work from the participants. In this article, we have introduced and discussed four categories of work health personnel perform to make sense of technology-mediated interaction.

Health personnel work to make sense of how to perform competently in technology-mediated interaction. They struggle to learn the basic technical skills, to define what type of communication this represents and to apply and adjust what they have learnt in their training within the new framework. It is unclear what frame (Goffman, 1974) the communication should be understood within, and health personnel negotiate the rules and expectations for how to perform in a competent manner in this setting. Further, health personnel negotiate how to relate to immediacy in technology-mediated interaction. Immediacy is experienced as something very different in technology-mediated interaction compared with face-to-face interaction, where you get immediate response, and health personnel negotiate how and what should be communicated at what time. The expected response time varies, even between different persons they communicate with, indicating that expected response time is influenced by normative expectations and user practices (Hutchby, 2003; Rettie, 2009).

The third category in our material concerns how health personnel deal with less access to social cues – or the need to reinvent social cues – in technology-mediated interaction. The reduced access to social cues can be perceived as a challenge, or an advantage, depending on what type of interaction the technology is supposed to facilitate. When the technology-mediated interaction is supposed to flow almost like informal face-to-face interaction, the reduced access to social cues represents a challenge for the participants. On the other hand, when the technology-mediated interaction represents a formal exchange of information, the reduced access to social cues is sometimes seen as an advantage, keeping the interaction focused and precise. Lastly, we have looked into the work involved to establish and maintain commitment in technology-mediated interaction. There is work needed from both participants in the interaction and from managers and others to facilitate participation and sustain commitment in technology-mediated interaction. This work includes how to deal with the problem of ‘free-riders,' and how to handle cooperating partners, who do not always commit to using the technology as expected.

We outline three main findings from our study. First, we argue that the introduction of technology-mediated interaction implies new frames for the interaction to be understood within (Goffman, 1974). This means that health personnel *work* to make sense of the new situations and participate in negotiations on meaning and implications for action down to the very smallest details – to put it in Goffman's words – ‘in a cold sweat' (Goffman, 1974, p. xii). Implications for technology introduction initiatives in health care include a need to acknowledge this work and give time for health personnel to understand the new situation and develop the new practices. Second, we argue that the introduction of technology-mediated interaction does not imply one new, defined form of interaction. Even within the frames of one technology, the outcome of communication and interaction can vary depending on the technology itself, user practices and expectations. Following this, our findings point towards the necessity of a complex hybrid of information and communication mediators in health care, meaning that technology introduction initiatives need to consider different types of technology for different types of communication they wish to facilitate. Third, we argue that the physical face-to-face meeting has some unique qualities (Goffman, 2005) that the participants in our study find it hard to replace, even if they commit to using the new technologies. This implies that when technology-mediated interaction is introduced, it is still necessary to include the possibility to meet face-to-face in some instances.

The introduction of mediating technologies redefines what is considered up-to-date, ‘good' health-care work. It challenges health personnel to change (some of) their work practices and moves, as a result, far beyond simple interventions aimed at making work more efficient. Do we see the contours of a new type of health personnel rising from these demands? A thorough understanding of how health personnel make sense of technology-mediated interaction is required in order to integrate these technologies in health-care practices in a proper way. This article makes a contribution in this respect, and further research should look into how health and care are being performed in increasingly new ways.

## Figures and Tables

**Table 1:** Interviewees projects A and B

	*Project A*	*Project B*
Public-health workers (nurses and assistants)	14	—
Home-care nurses	—	23
GPs	—	11
Secretaries and project managers	—	9
Total	14	43
